# The Use of Recombinant Feline Interferon Omega Therapy as an Immune-Modulator in Cats Naturally Infected with Feline Immunodeficiency Virus: New Perspectives

**DOI:** 10.3390/vetsci3040032

**Published:** 2016-10-27

**Authors:** Rodolfo Oliveira Leal, Solange Gil

**Affiliations:** 1 Centre Hospitalier Vétérinaire Fregis, 43 Av. Aristide Briand, Arcueil 94110, France; 2 Centro de Investigação Interdisciplinar em Sanidade Animal (CIISA), Faculdade de Medicina, Veterinária, University of Lisbon (ULisbon), Av. Universidade Técnica, Lisbon 1300-477, Portugal; solange@fmv.ulisboa.pt

**Keywords:** feline, interferon therapy, recombinant-feline interferon omega, immune modulation, feline immunodeficiency virus

## Abstract

Type I interferons (IFNs) are well-known cytokines that, among their main functions, are key components of the host immune response against viral infections. Due to its immune modulation properties, they are commonly used in the therapeutic approach of various retroviral infections, namely human immunodeficiency virus (HIV) and feline immunodeficiency virus (FIV). In HIV infection, it has been shown that IFN therapy limits early viral replication, particularly useful on post-exposure prophylaxis. In veterinary medicine, recombinant feline interferon omega (rFeIFN-ω) was the first interferon licensed for use in cats. Several studies have recently shown that this compound seems to stimulate the innate immunity, decreasing clinical signs and co-infections in naturally FIV-infected cats. More than summarizing the main conclusions about rFeIFN-ω in cats, this review emphasizes the immune-modulation properties of IFN therapy, opening new perspectives for its use in retroviral infections. Either in FIV-infected cats or in HIV individuals, type I IFNs seem to induce an innate immune-modulation and should not be overlooked as a therapeutic option in retroviral infections.

## Mini-Review

In clinical practice, the initial therapeutic approach in cats suspected of retroviral infections is always supportive and symptomatic. When the diagnosis of retroviral infection is established, antivirals and immune modulators can be considered on short- and long-term management. Taking into account that most of these drugs are licensed for use in humans, there is a lack of well-controlled clinical trials in cats and their efficacy is not entirely clear [1,2].

Nowadays, there are no antivirals licensed for use in veterinary medicine with the exception of several immune modulators, which have concurrent antiviral properties. Therefore, all the truly antiviral compounds used in dogs and cats are those licensed for use in humans, namely in HIV therapies [1,2,3]. On the other hand, interestingly, the administration of antivirals in cats has been frequently documented due to the fact that FIV-infected cats are commonly used as HIV experimental models. Even if antivirals have shown a low efficacy in cats and can induce significant toxic effects, several of them are used on the management of retroviral infections [1,2,4]. Among them, the RT inhibitors/nucleoside analogues are the most common. The majority are nucleoside analogues which, acting as anti-metabolites, are “false substrates” that bind to RT enzyme and block its activity [4]. Other antivirals that can be used in retroviral infections are antagonists/homologous of receptors, namely byciclams such as plerixafor and AMD 3100, which bind either to viruses (homologous) or to cell-receptors (antagonists), inhibiting the virus–cell interaction. With the exception of several drugs, these compounds are strongly selective for HIV, meaning that they are not used in veterinary medicine [4].

Taking part in the group of “immune therapy”, by definition, immune modulators are compounds that interfere with the immune system. They are commonly used in different clinical situations, particularly in canine and feline viral infections. It is believed that immune modulators restore several functions of the immune system, allowing a better management of opportunistic infections and a better clinical recovery. Some of these compounds can even have a direct antiviral effect [4]. In between the well-known immune modulators, interferons (IFNs) will be further discussed, mainly due to its current use in retroviral infections.

Interferons (IFNs) are key components of the host immune system, being particularly relevant in viral infections [5]. The large family of IFNs can be divided into different types. Type I IFNs are the most studied ones as they are commonly used for therapeutic purposes. Among their major functions, type I IFNs increase and sensitize the immune system towards the microbial recognition [6], establishing an important link between innate and acquired immunity [7]. Furthermore, they have different anti-viral properties, blocking viral replication and inducing apoptosis of infected cells [8,9,10,11].

In human immunodeficiency virus (HIV), the therapeutic use of IFNs has been assessed in various in vitro and in vivo studies. In vitro, it was proven that IFNs are able to restrict HIV replication inducing an antiviral state and preventing viral infection [12,13]. In vivo, despite the HIV’s evasion mechanisms, IFNs seem to play an important protective role against HIV infection in seronegative individuals exposed to the virus [14,15]. Moreover, its therapeutic administration showed a durable suppression of viral replication and decreased viral integration following anti-retroviral therapy in HIV individuals [16]. Even if further studies are needed to investigate the therapeutic effects of IFN in HIV, its protective role on the early stages of infection aims to consider that IFN therapy seems particularly useful on post-exposure prophylaxis [15].

Not only in humans but also in feline medicine, the use of type I IFNs as immune modulation therapy is common, particularly in retroviral infections [17,18,19].

Still used in several countries, HuIFN-α was the first interferon administered in cats, despite the fact that it is only licensed for human medicine [4]. Among their major functions, it was shown that HuIFN-α inhibits oncogenic mutations induced by retroviruses, and it restrains viral nucleic acid synthesis and suppress protein production. Due to these anti-viral and immune modulation properties, HuIFN-α is used with various human diseases such as myeloid leukemia, papilomatosis, and HIV [4]. For the same reasons, HuIFN-α has been also used in feline medicine, namely in FIV, FeLV, FHV-1, FCV, and FCoV viral infections [20,21,22,23]. In spite of its short-term effects, particularly on clinical improvement and the increase in survival time, the development of neutralizing antibodies several weeks after therapy makes HuIFN-α ineffective for long-term immune modulation therapy in cats [4,19,21]. This problem was bypassed by the more recent release of recombinant feline interferon omega (rFeIFN-ω).

RFeIFN-ω is the first interferon compound licensed for use in veterinary medicine. Specifically in cats, this product is approved for use in retroviral infections, namely feline immunodeficiency virus (FIV) and feline leukemia virus (FeLV). According to the manufacturer’s instructions and license, it should be administered in three cycles of five daily subcutaneous injections of 1 MU/kg, beginning respectively on days 0, 14, and 60. Despite the fact that it was licensed a few years ago, only a few studies supported its clinical benefits, particularly in retroviral infections. The first study, which described its clinical application dates from 2004 and reported that treated feline leukemia virus (FeLV) and FIV/FeLV co-infected cats, showed a significant clinical improvement and an increased survival time [18]. More recently, in 2011, another research group reinforced that rFeIFN-ω improves clinical signs and hematologic parameters in retrovirally infected cats. However, according to this study, it does not change hypergammaglobulinemia, proviral load, or viremia, suggesting an overall effect mainly on the innate immune reaction rather than on the acquired immunity [17]. This means that rFeIFN-ω does not seem to have an anti-viral effect in vivo, and the observed clinical improvement must be related to a potential immune modulation of the innate immune system [17]. Moreover, in contrast to in vitro studies [24], the anti-viral in vivo action of the rFeIFN-ω licensed protocol towards FIV and FeLV was found to be negligible according to these authors [17]. These points highlight the need of further studies in order to clarify the mechanisms of action of rFeIFN-ω.

The authors tried to extend the current knowledge about this compound in order to support and clarify the evident clinical improvement previously described [17,18]. Experimental work relied on two distinct clinical trials [25,26,27]. One referred to 16 retrovirally infected cats (7 FIV, 6 FeLV, and 3 co-infected cats) treated with rFeIFN-ω licensed subcutaneous protocol, while the other described an alternative oral rFeIFN-ω protocol, administered to 11 FIV-infected cats [25,26]. Both protocols were compared in an immunological and virological perspective [25,26,27].

Firstly, the effect of rFeIFN-ω licensed protocol in cats living in an animal shelter was evaluated, assessing clinical improvement and monitoring concurrent viral excretion (namely herpesvirus, calicivirus, and coronavirus) [26]. A previous study in cats treated with HuIFN-α documented that the induced clinical improvement could be potentially related to a recovery of serious opportunistic infections [21]. In order to assess whether rFeIFN-ω had the same properties, different hematology and biochemistry parameters were monitored, and a score-scale system that allowed the evaluation of clinical improvement in treated cats was validated. In agreement with the two previous referred studies [17,18], a significant clinical improvement in treated animals without relevant changes on hematology and biochemistry profiles was reported [26]. Concerning these results, rFeIFNω seems to be able to contribute to the management of this reality by improving clinical signs and decreasing concurrent viral excretion. Therefore, rFeIFNω must be considered as an effective immune-modulator therapy for use in shelter medicine where the prevalence of concomitant infections is higher.

In further studies, the influence of rFeIFN-ω in the acute phase reaction was investigated [28]. In human medicine, several studies have described its increase in HIV-positive patients [29,30], even after immunomodulation therapy with exogenous IL-2 [31]. Despite the similarity between HIV and FIV [2,32], the CRP behavior in FIV-positive cats undergoing immune-modulation therapy remains unknown. Following previous conclusions suggesting that this compound acts on the innate response rather than on the acquired immune system, its effect on acute phase proteins (APPs) was evaluated [17]. Taking into account that APPs are one of the key components of the innate immune system, they seemed reasonable predictors of an innate immune-stimulation [33]. Authors showed that APPs significantly increased in cats treated with the licensed rFeIFN-ω protocol [28]. Despite the fact that APPs usually increase in different situations such as chronic infection and severe inflammation [33,34], in this study, their increment was concomitant with the described clinical improvement and reduction in opportunistic infections. This work showed that the APP increment might be beneficial in retrovirally infected cats, confirming that they can be a reasonable indicator of a potentiated innate immune response. Being the first work documenting the effect of rFeIFN-ω on APP profiles, this study reinforces the action of this compound on the innate immune system, helping to clarify the mechanisms of action of the licensed protocol [28]. In order to explore in greater detail these conclusions, the evaluation of other innate immunity biomarkers (namely Mx protein) would be useful. Although its use in feline medicine is scarce, Mx protein is a specific type I IFN biomarker [8,35]; hence, its production is directly related with the activation of a type I IFN signal transduction pathway. In this sense, from the authors’ point of view, further studies are needed to strengthen these conclusions and to assess whether Mx protein can be a reasonable biomarker of innate immunity in cats under rFeIFN-ω therapy.

Recognizing that, in clinical practice, rFeIFN-ω is very often a cost-limiting therapy, the use of an alternative oral protocol in FIV-infected cats has also been documented [25]. In fact, different authors reported the clinical benefits of oral low dose HuIFN-α protocols in viral infections such as FIV [19,21]. After the release of rFeIFN-ω, several clinical trials had also been conducted in specific-pathogen free cats and calicivirus-positive cats in order to study the immune-modulation properties of oral rFeIFN-ω [8,36]. However, no studies have documented its use in retrovirally infected cats, particularly in FIV-infected ones. In this sense, an oral rFeIFN-ω protocol was developed and administered to 11 client-owned FIV-infected cats [25]. The oral dose was based on the referred previous studies and was decided according to the manufacturer’s previous trials [8,36]. The oral protocol was 10-fold lower that the licensed subcutaneous protocol, and it was given orally instead of subcutaneously and for a longer period of time (90 continuous days). Following the same methodology of previous studies, the clinical improvement, concurrent viral excretion, APP profiles, and different hematology and biochemistry parameters in FIV-infected cats treated with the oral protocol were assessed. Similarly to what was observed for the licensed protocol, cats treated with oral rFeIFN-ω showed a significant clinical improvement without remarkable changes in hematology and biochemistry profiles, which were within the reference range in the majority of the treated cats [25]. Conversely, mainly due to the fact that client-owned cats were less prone to opportunistic infections, concurrent viral excretion was very low and did not change with the applied protocol. Additionally, APP profiles did not change in cats treated with oral rFeIFN-ω, meaning that the two distinct protocols seem to act differently. Taking APPs as biomarkers of the innate immune response, it seems reasonable to say that innate immune reaction does not seem to be potentiated in the same way as observed in the licensed protocol. However, as interferons have pleotropic effects and APPs are only a part of the innate immune response, it is overspeculative to assume that oral rFeIFN-ω does not interfere with innate immunity. Even if APPs did not change, overall results showed an important clinical improvement and reinforced the potential extra-label use of rFeIFN-ω in an oral continuous low-dose protocol.

For a second time, the effect of both protocols of rFeIFN-ω on cytokine profile, viremia, and proviral load was evaluated [27]. Only one study had previously reported that the licensed protocol does not change viremia or proviral load in treated FIV-infected cats, suggesting that this compound may not act on acquired immunity [17]. In agreement with these results, authors concluded that viremia did not change in the group of FIV-infected cats treated with the licensed protocol [27]. However, in opposition to what was previously reported, a significant increase on proviral load was reported and correlated to a relative increase of lymphocytes cell count (even within the normal range) [27]. In this study, viremia and proviral load changes in FIV-infected cats treated with oral rFeIFN-ω were also evaluated. As expected, no changes were obtained in either parameter, which reinforced the previous suggestion that, independently of the administered protocol, the rFeIFN-ω’s anti-viral effect in vivo is negligible. At this point, it was concluded that rFeIFN-ω licensed protocol induced a decrease in concurrent viral infections, even if there were no true changes in FIV viral load. On the other hand, in the oral protocol, albeit no changes on the viral load and concurrent viral infections, there was a significant clinical improvement in treated cats. Therefore, even if the antiviral properties against FIV seem negligible in both protocols, the fact that there was a decrease in concurrent viral infections in cats treated with the licensed protocol raises the question of whether there is a true antiviral effect towards other viruses or whether this is the result of an overall stimulation of the innate and acquired immune system. From the authors’ point of view, it is overspeculative to state that rFeIFN-ω does not act on the acquired immune system only based on viremia and proviral load. Therefore, an evaluation of cytokine profiles in these animals seemed essential to fully understand the actions of rFeIFN-ω.

In order to reinforce the previous suggestions that rFeIFN-ω does not affect the acquired immune system of FIV-infected cats, mRNA expression and concurrent plasma levels of various cytokines were monitored using biological samples from the two groups of FIV-infected cats treated with either subcutaneous or oral rFEIFN-ω protocols [27]. Despite its pleiotropic effect, variations of Th-1 and Th-2 responses were assessed based on different measured cytokines. Results showed that Th-1 and Th-2 responses did not significantly change in either protocol, which supported the previous suggestions that rFeIFN-ω does not strongly affect the acquired immune system. Among the measured cytokines, only IL-6 (a pro-inflammatory cytokine involved in different immune pathways and particularly in the innate immune response) significantly changed in both groups. In fact, in cats treated with the licensed protocol, IL-6 plasma levels were significantly reduced, whilst its respective mRNA expression showed a decreasing tendency that was not statistically significant. On the other hand, in cats treated with oral rFEIFN-ω, IL-6 plasma levels did not change, but the concurrent mRNA expression significantly decreased. All in all, it was documented that IL-6 production is affected in both protocols, meaning that rFeIFN-ω has anti-inflammatory properties. Moreover, considering that plasma changes were only significant in cats treated with the licensed protocol, it seems reasonable to state that this higher pulsate therapeutic scheme is more efficient in reducing the pro-inflammatory stimuli than the continuous low dose therapy.

These were the first studies exploring the effect of rFeIFN-ω in different biomarkers of the immune system. However, there are still many points to investigate in order to explore the whole potential of this compound in retroviral infections. In FIV-infected cats, only two protocols were assessed. Following the same research line, it would be interesting to reinforce these conclusions with pharmacokinetic and pharmacodynamic studies. In the subcutaneous protocol, it would be useful to document the effect of lower doses but in longer therapy cycles and vice-versa, in order to correlate the therapy requirements with clinical and virological conditions. For the oral group, it would also be interesting to assess the effect of intermediate higher doses for shorter periods and also to extend the protocol to FeLV-infected cats. In spite of the different physiopathology and the aggressive clinical portrait, it would be interesting to assess the effect of the suggested oral rFeIFN-ω in these animals. According to the authors’ point of view, it is unlikely that such a lower oral dose will have the same effect on clinical improvement of FeLV-infected cats since these animals are usually more symptomatic and in worse overall clinical condition than FIV-infected animals. Consequently, the higher subcutaneous protocol will always be preferable in these cats.

As previously stated, several studies have shown that IFNs can be beneficial in the early stages of HIV infection [15]. Recognizing that these results concern naturally infected cats in which the time of infection is unknown, these findings are in some way in agreement with these HIV studies as they indirectly reflect an innate immune-modulation and decrease in clinical signs and co-infections. Compared with exploring the full potential of each route and dose, it would be interesting to study a combination of protocols to be used according to different clinical presentations. Although there are no studies assessing a possible combination of both protocols, we think that they can be reasonably associated, as both therapies are well documented and do not show important adverse effects. For instance, in symptomatic animals, an initial subcutaneous protocol followed by an oral continuous lower-dose therapy can now be recommended. From the authors’ point of view, this seems a reasonable approach that sooner or later can be considered in routine clinical practice.

Also regarding the follow-up and life span, studies are warranted. In fact, some authors have tried to perform monthly to trimester follow-ups after the end of therapy. In both groups, it was unsuccessful. In the animal shelter, clinical evaluations after therapy were unreasonable once the income and outcome of cats impaired correct conclusions. Regarding client-owned cats, it was only correctly achieved in a small number of treated animals (unpublished data). In fact, the individual oral therapy for each cat was free of charge and comprised a total of 90 daily doses that were partially given to the owners at each evaluation time point. After the end of the therapy, the monitoring was unpractical once the majority of owners were unavailable to pursuit with monthly follow-ups. Consequently, a structured prospective study would be interesting in order to evaluate not only the long-term benefits of therapy but also any effect on the mean life span of FIV-infected cats.

These studies innovated in the extension of the main therapeutic properties of the licensed rFeIFN-ω protocol in naturally retrovirally infected cats. It is now documented that it must be used not only in client-owned symptomatic cats but also in animals living in catteries or shelters where opportunistic infections are problematic. Although without a truly effect on the cytokine profile, this compound induces a significant clinical improvement and seems to reduce the pro-inflammatory stimuli. Particularly, for FIV-infected cats, this work presents a new oral rFeIFN-ω protocol, which was successfully tested and validated. Although it induces a significant clinical improvement, its overall action as an immune modulator is less than the subcutaneous protocol. In fact, it slightly decreases the pro-inflammatory stimuli without affecting the acquired immunity, or even other parameters of the innate response such as acute phase proteins. Therefore, whilst the high pulsate subcutaneous protocol is strongly recommended for symptomatic FIV-infected cats, this lower continuous oral protocol is a good alternative for cats presenting mild clinical signs, for cases where cost limits the use of licensed protocol or even for cats, which previously received subcutaneous rFeIFN-ω and require continuous immune modulation therapy. These studies did not contribute to a better understanding of rFeIFN-ω as much as they explored its immune modulation properties and validated a new oral protocol, which can be included in future FIV-guidelines.

In summary, this review reinforces the beneficial properties of IFN therapy opening new perspectives for its use in retroviral infections. Even if rFeIFN-ω is a species-specific compound, these conclusions can be extrapolated to a larger perspective. Either in FIV-infected cats or in HIV individuals, type I IFNs seem to induce an innate immune-modulation and should not be overlooked as a therapeutic option in retroviral infections.
